# Proteomic analysis of nemaline myopathy in infants reveals distinct common dysregulated proteins and cellular pathways

**DOI:** 10.3389/fneur.2025.1661747

**Published:** 2025-10-03

**Authors:** Carola Hedberg-Oldfors, Ali Zeki Bedir, Kittichate Visuttijai, Eva Michael, Anders Oldfors

**Affiliations:** ^1^Department of Laboratory Medicine, University of Gothenburg, Gothenburg, Sweden; ^2^Cizre State Hospital, Sirnak, Türkiye; ^3^Department of Pediatrics, University of Gothenburg, Gothenburg, Sweden

**Keywords:** nemaline myopathy, pathology, proteomics, dysregulated proteins, proteinsynthesis, protein degradation, glycolysis

## Abstract

**Background:**

Nemaline myopathy is a rare congenital muscle disorder characterized by the presence of nemaline rods, protein aggregates, in muscle fibers. Pathogenic variants in several genes, most commonly *NEB* and *ACTA1*, which encode thin filament proteins of the sarcomere, have been implicated in its etiology. Currently, there is no cure for nemaline myopathy, underscoring the need to identify disease-modifying targets for therapeutic development.

**Methods:**

In this study, we employed quantitative nanoscale liquid chromatography–tandem mass spectrometry (LC-MS^3^) with labeled protein analysis on muscle tissue from five normal controls and seven infants diagnosed with nemaline myopathy due to *NEB* or *ACTA1* pathogenic variants.

**Results:**

We identified and quantified 4,846 proteins across all samples, with 183 proteins showing significant dysregulation. Protein–protein interaction analysis revealed nine upregulated, muscle-specific proteins: NRAP, FBXO40, TRIM63, TRIM54, ALPK3, XIRP1, ANKRD2, LMOD2, and CSRP3. Further pathway analysis indicated upregulation of protein synthesis and proteasomal degradation processes, alongside downregulation of glycolysis. Notably, the dysregulated proteins and pathways were consistent across both genetic subtypes, suggesting shared molecular mechanisms underlying the disease.

**Conclusion:**

This proteomic profiling study has identified key dysregulated proteins and pathways in infantile nemaline myopathy. These findings advance our understanding of the disease’s molecular basis and highlight candidate targets for future therapeutic intervention.

## 1 Introduction

Nemaline myopathy is one of the most common forms of congenital myopathy and is characterized by the presence of numerous small protein aggregates named nemaline rods in the muscle fibers (1). Nemaline myopathies have traditionally been classified into different types based on their clinical presentation (2, 3). At least 12 different genes have been associated with nemaline myopathy, *NEB* encoding nebulin and *ACTA1* encoding alpha-actin are the most prevalent (4). Nemaline myopathy caused by pathogenic *NEB* variants usually show recessive inheritance while *ACTA1* associated nemaline myopathy is usually caused by dominant, mostly *de novo*, variants (3). All nemaline myopathies seem to be associated with proteins involved in the structure and function of the thin filaments of the sarcomere (3). In spite of the many genes involved there are morphological similarities with regard to the common pathological hallmark, the nemaline rods, but there are also differences with regard to fiber type composition, severity of morphological changes as well as age-related changes (5, 6). Currently, there are no therapies available for nemaline myopathy. Proteomic analysis of affected muscle tissue is an emerging research field that may, together with genomics, help identify dysregulated proteins and protein networks to reveal pathobiological mechanisms, novel biomarkers, and identify potential therapeutic interventions in neuromuscular disorders (7). In this study, we used a proteomic approach to identify dysregulated proteins and altered cellular pathways in muscle at infancy of the two major genetic forms of nemaline myopathy with similar clinical phenotype and muscle biopsy histopathology. This work identified several dysregulated proteins, which are potential targets to treat nemaline myopathy.

## 2 Materials and methods

### 2.1 Material

Muscle biopsy specimens from seven infant patients diagnosed with intermediate congenital nemaline myopathy (2) were included in this study (Table 1). The mean age of the patients at biopsy was 5.4 months (range, 2–19 months). Three patients had *de novo* dominant *ACTA1* variants, and 4 patients were compound heterozygous for *NEB* variants (Table 1). Skeletal muscle controls included individuals with normal muscle biopsies who had been investigated for a possible mitochondrial disease, but in whom the clinical, biochemical and pathological investigations excluded muscle disease. The mean age of the controls at biopsy was 3.6 months (range, 1–9 months).

**Table 1:** Genetic information for the patients included in this study.

Patients	Gene	DNA change	Amino acid change	Inheritance^#^	gnomAD Allele count	CADD score	Clin-Var	HGMD	Reported*
P1	ACTA1	c.809G > A	p. Gly270Asp	AD (het) De novo	0	32	P/LP	DM (6)	(6)
P2	ACTA1	c.1123A > G	p. Lys375Glu	AD (het) De novo	0	32	LP	DM (6)	(6)
P3	ACTA1	c.553C > A	p. Arg185Ser	AD (het) De novo	0	28	P/LP	DM (37)	This paper
P4	NEB	c.20001C > G c.24502_24503dupTT	p. Asp6667Glu p. Leu8168fs*13	AR Comp. het	0 0	23.7 -	- P	- -	This paper
P5	NEB	c.23140C > T c.25183C > T	p. Arg7749* p. Arg8430*	AR Comp. het	2/1599508 4/1609356	52 51	P P/LP	DM (38) DM?	This paper
P6	NEB	c.4300-3C > A c.6937C > T	Splice? p.(?) p. Arg2313*	AR Comp. het	0 0	- -	- P	- DM (39)	This paper
P7	NEB	c.3255 + 1G > T c.9859C > T	Splice? p.(?) p. Gln3287*	AR Comp. het	30/1343354 0	33 43	P/LP -	DM (40) -	This paper

ACTA1, M_001100.4; NEB, NM_001271208.2; ^#^, confirmed by segregation analysis; gnomAD version v4.1.0; HGMD, Human Mutation Database Professional 2025.1 (Qiagen); * patients previously reported. P1-P7; patients 1–7; AD; autosomal dominant, AR; autosomal recessive, het; heterozygous, Comp. het; compound heterozygous; P; Pathogenic, LP; Likely pathogenic, DM; disease causing.

This study was conducted according to the Declaration of Helsinki and approved by the Swedish Ethical Review Authority, approval number 2022–00026-01.

### 2.2 Muscle biopsy and histopathological investigations

For histopathological investigations, open biopsy of the vastus lateralis muscle was performed. Specimens were mounted on cork plates and snap frozen in isopentane in liquid nitrogen. One specimen in each case was fixed in buffered glutaraldehyde for electron microscopy. Routine methods were applied for morphological and enzyme histochemical investigations (8). Fiber typing was assessed by myofibrillar ATPase staining or myosin heavy chain immunohistochemistry (8, 9).

### 2.3 Proteomic investigations

Skeletal muscle protein extracts from 7 patients and 5 normal controls were prepared from fresh frozen muscle biopsies. For quantitative analysis the proteins were labeled using TMTpro 18-plex isobaric mass tagging reagents (Thermo Fischer Scientific) and analyzed by nanoscale liquid chromatography–tandem mass spectrometry (LC-MS^3^) according to details described in Supplementary material. Raw files were processed and analyzed with Proteome Discoverer against UniProt Swiss-Prot *Homo Sapiens* using Sequest as a search engine. The gene symbols are used throughout the manuscript to describe the encoded proteins. Protein data were partly analyzed with the software Omics Playground (BigOmicsAnalytics, v3.5.24) (10). To identify differentially expressed proteins, the data was log2-transformed, and then, for each protein, log2 fold change (log2FC) and *p*-values were computed using Welch’s t-test for patients versus controls. To control for multiple comparisons, the Benjamini-Hochberg method was used to adjust the p-values, and proteins with a false discovery rate (FDR) less than 0.05 were considered significant.

Pathways analysis and protein–protein interaction analysis were performed by applying different search tools and web resources such as ProteoMap website^1^ (11) and STRING website^2^ (12).

## 3 Results

### 3.1 Histopathological investigation

**Figure 1:**
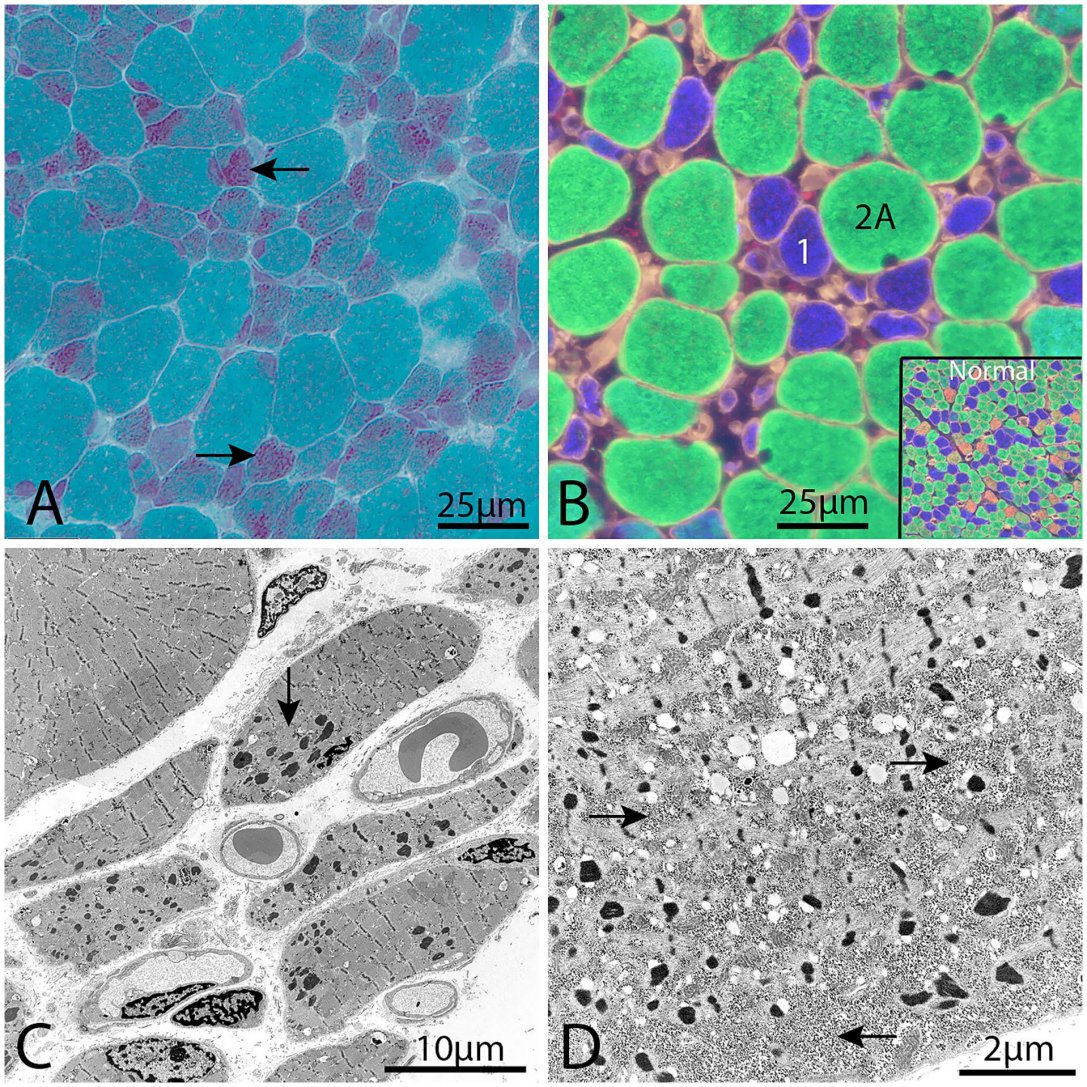
Representative example of a muscle biopsy of a patient with nemaline myopathy caused by *NEB* variants. **(A)** There is an increased fiber size variability and the majority of fibers show nemaline rods, especially the small fibers (arrows), Gomori trichrome. **(B)** Immunofluorescence of myosin heavy chain isoforms (see Material and methods). The small fibers are type 1 fibers (blue) and the large fibers are type 2A fibers (green). No type 2X fibers (red) are present, compared with inset showing normal muscles. **(C)** Electron micrograph showing nemaline rods and disrupted myofibrils in the muscle fibers. **(D)** Electron micrograph showing glycogen accumulation (arrows) between myofibrils and nemaline rods in a muscle fiber.

**Figure 2:**
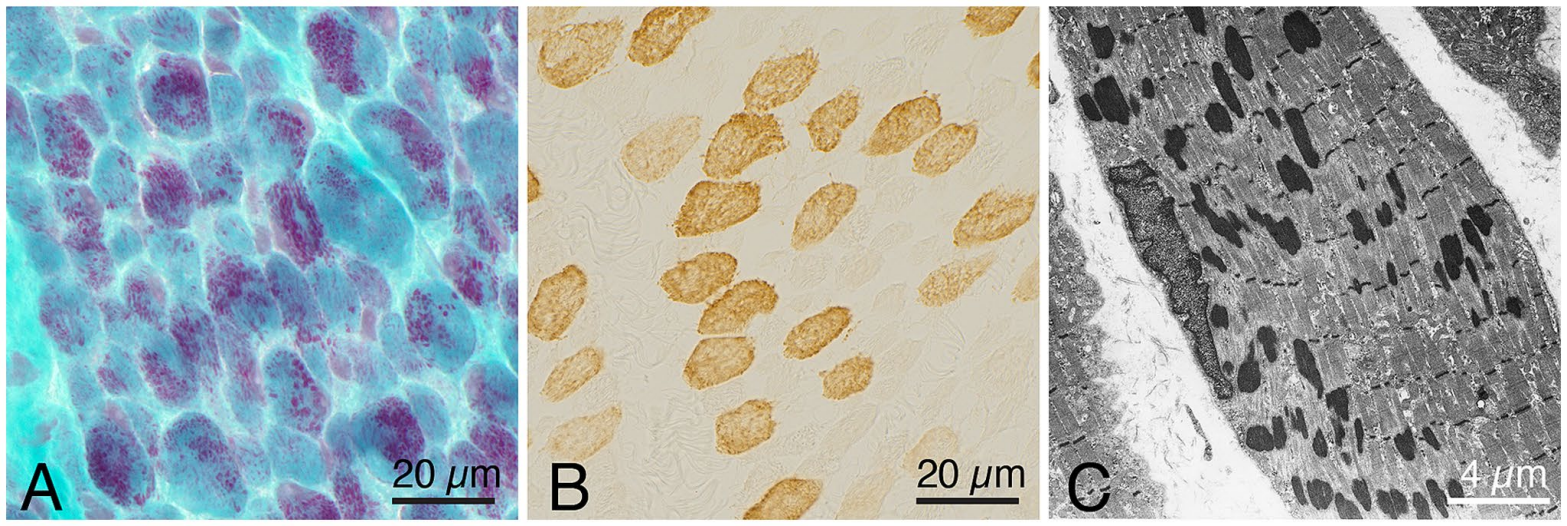
Representative example of a muscle biopsy of a patient with nemaline myopathy caused by an *ACTA1* variant. **(A)** There is an increased fiber size variability, and the majority of fibers show nemaline rods, Gomori trichrome. **(B)** Immunohistochemistray of myosin heavy chain fast type, showing presence of both type 1 (unstained) and type 2 (stained) fibers. **(C)** Electron micrograph showing nemaline rods in a muscle fiber.

Histopathological investigation showed a similar pattern of nemaline myopathy in all cases, (Figures 1, 2). Nemaline rods were seen in most fibers but mainly in small type 1 fibers. Both type 1 and type 2 fibers were present in all cases, with some variability of fiber type predominance, and also in different regions of the same biopsy. In addition to nemaline rods, other structural alterations of the myofibrils were also frequent.

### 3.2 Proteomic investigation

The proteomic profiling was performed using quantitative analysis based on nanoscale liquid chromatography coupled to tandem mass tag labeling (TMT) and tandem mass spectrometry (LC-MS^3^). From the basic analysis of the proteomic data, 4,846 proteins were identified in all samples and quantified, of which 183 proteins were significantly and differently expressed in the nemaline myopathy patients compared to the control group [adjusted *p*-value (FDR) < 0.05]: 112 proteins were increased and 71 were decreased (Supplementary Table 1). Of the 183 proteins, 43 proteins were > 2 times up- or down-regulated (Table 2).

**Table 2:** The most significantly up-and down-regulated proteins.

Protein expression	Gene symbol	Protein accession	Description	log2FC	FDR
Up-regulated	ASXL2	Q76L83	ASXL transcriptional regulator 2	2.226	0.027
	TRIM63	Q969Q1	tripartite motif containing 63	2.201	0.016
	ANKRD2	O15084	ankyrin repeat domain 2	1.906	0.032
	INPP4B	O15327	inositol polyphosphate-4-phosphatase type II B	1.899	0,027
	CYP2J2	P51589	cytochrome P450 family 2 subfamily J member 2	1.585	0.016
	XIRP1	Q702N8	xin actin binding repeat containing 1	1.481	0.044
	HOMER2	Q9NSB8	homer scaffold protein 2	1.401	0.027
	ALPK3	Q96L96	alpha kinase 3	1.365	0.016
	MAP3K7CL	P57077	MAP3K7 C-terminal like	1.332	0.031
	ICMT	O60725	isoprenylcysteine carboxyl methyltransferase	1.266	0.045
	KLHL21	Q9UJP4	kelch like family member 21	1.246	0.028
	OBSL1	O75147	obscurin like cytoskeletal adaptor 1	1.226	0.027
	CSRP3	P50461	cysteine and glycine rich protein 3	1.157	0.034
	LMOD2	Q6P5Q4	leiomodin 2	1.144	0.028
	SLMAP	Q14BN4	sarcolemma associated protein	1.137	0.028
	TM7SF2	O76062	transmembrane 7 superfamily member 2	1.125	0.027
	FBXO40	Q9UH90	F-box protein 40	1.051	0.039
	CAMK2D	Q13557	calcium/calmodulin dependent protein kinase II delta	1.029	0.028
	DIAPH1	O60610	diaphanous related formin 1	1.002	0.028
Down-regulated	PKM	P14618	pyruvate kinase M1/2	−1.010	0.028
	GPD1	P21695	glycerol-3-phosphate dehydrogenase 1	−1.010	0.044
	PGM1	P36871	phosphoglucomutase 1	−1.018	0.028
	ADSS1	Q8N142	adenylosuccinate synthase 1	−1.025	0.032
	PLCL1	Q15111	phospholipase C like 1 (inactive)	−1.033	0.028
	WDR11	Q9BZH6	WD repeat domain 11	−1.073	0.027
	GPD2	P43304	glycerol-3-phosphate dehydrogenase 2	−1.074	0.025
	AK1	P00568	adenylate kinase 1	−1.091	0.037
	CRADD	P78560	Death domain-containing protein CRADD	−1.107	0.034
	TLE1	Q04724	TLE family member 1, transcriptional corepressor	−1.173	0.028
	CA14	Q9ULX7	carbonic anhydrase 14	−1.175	0.034
	EGFLAM	Q63HQ2	EGF-like, fibronectin type-III and laminin G-like domains	−1.209	0.027
	CMBL	Q96DG6	carboxymethylenebutenolidase homolog	−1.213	0.027
	TPPP	Q9BW30	tubulin polymerization promoting protein	−1.330	0.031
	IGFBP5	P24593	insulin like growth factor binding protein 5	−1.336	0.049
	DBI	P07108	diazepam binding inhibitor, acyl-CoA binding protein	−1.396	0.027
	BCL2	P10415	BCL2 apoptosis regulator	−1.478	0,027
	ART3	Q13508	ADP-ribosyltransferase 3 (inactive)	−1.503	0.028
	ACADL	P28330	acyl-CoA dehydrogenase long chain	−1.573	0.032
	MYH4	Q9Y623	myosin heavy chain 4	−1.738	0.028
	PTER	Q96BW5	phosphotriesterase related	−1.790	0.027
	RBM20	Q5T481	RNA binding motif protein 20	−2.086	0.016
	GJA8	P48165	gap junction protein alpha 8	−3.815	0.027
	MYH1	P12882	myosin heavy chain 1	−3.980	0.027

FDR < 0.05, Log2FC < −1 or >1.

**Figure 3:**
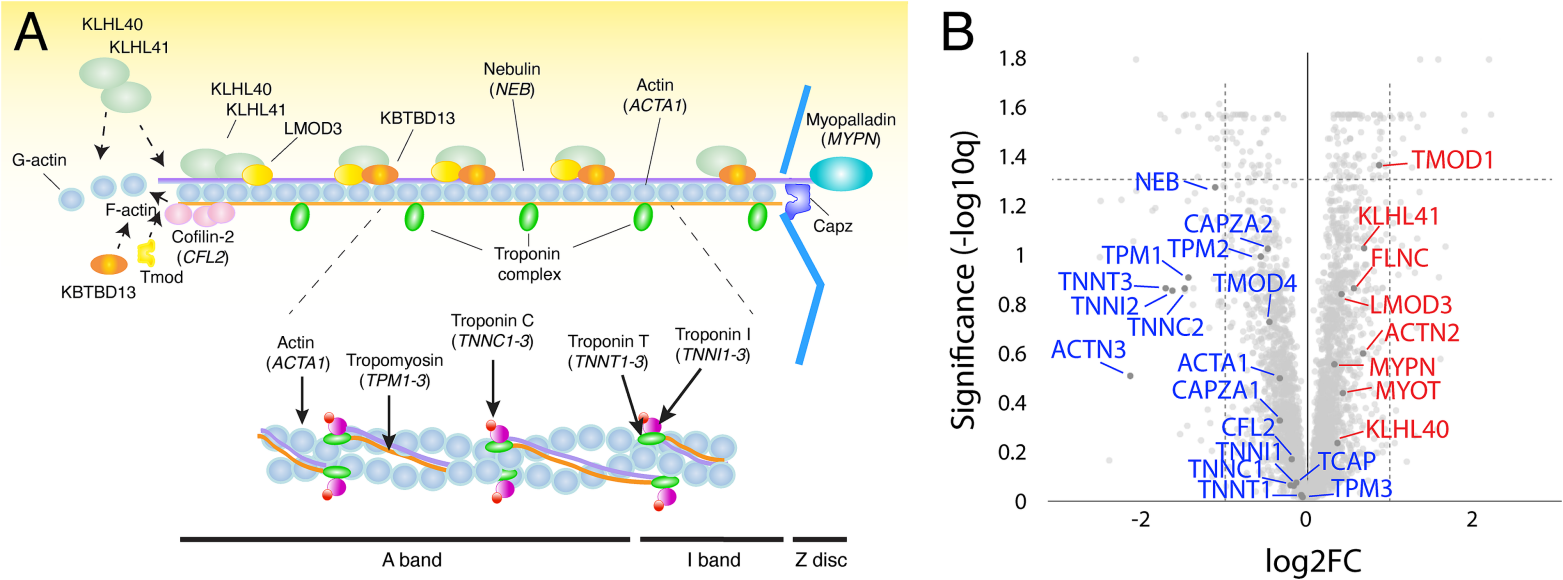
**(A)** Schematic illustration of the muscle thin filament in the sarcomere, including the 12 major nemaline myopathy-associated proteins. **(B)** Volcano plot with thin filament proteins and including the proteins associated with nemaline myopathy (in blue, down-regulated; in red, up-regulated). Proteins are given by their gene symbols.

Proteomic investigation of the thin filament proteins including, the 12 nemaline myopathy associated proteins revealed for most proteins non-significant changes (Figures 3A,B). The only significantly upregulated protein was tropomodulin, which is involved in the regulation of thin filament length (13). The expression of proteins that were mutated in our patients, alpha-actin (ACTA1) and nebulin (NEB), were not significantly altered. Alpha-actinin-2 (ACTN2), a Z-disc protein and immunohistochemical hallmark of nemaline rods, was neither significantly upregulated.

**Figure 4:**
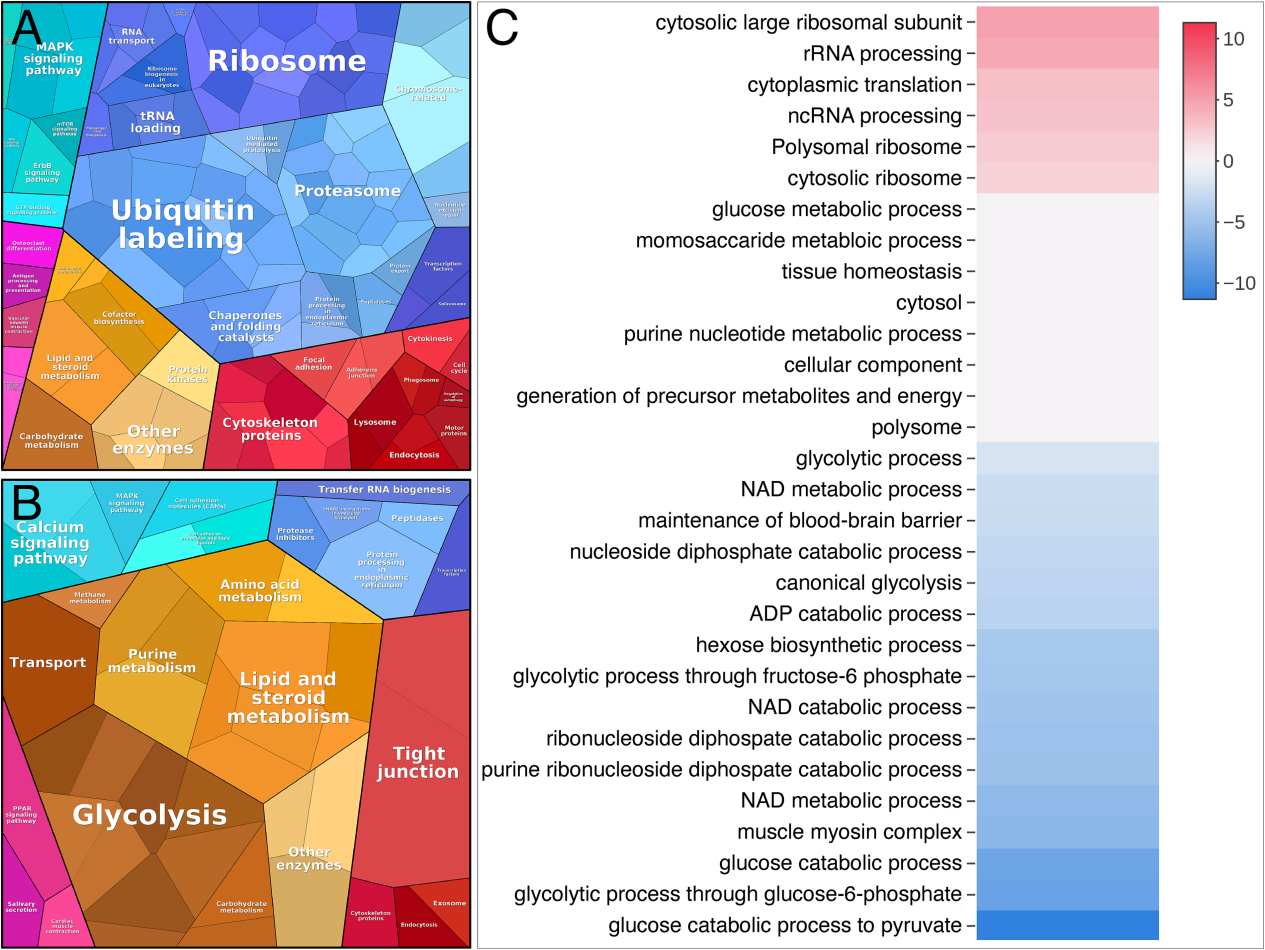
Proteome analysis visualized by Proteomap and Activation matrix analyses in patients compared to controls. **(A)** Proteomap showing that many up-regulated proteins are involved in ubiquitination, proteasomes and ribosomes. **(B)** Proteomap showing that many down-regulated proteins are involved in glycolysis. **(C)** Activation matrix (BigOmics Analytics) showing detected GO terms that are consistently up- or down-regulated, and are colored according to their upregulation (red) or downregulation (blue).

A gene ontology (GO-term) based in silico analysis of dysregulated proteins was performed to elucidate the biological processes and subcellular compartments affected in patients with nemaline myopathy. The result of this overall proteomic profiling indicated some major alterations from the normal muscle. These included signs of increased protein turnover, as evidenced by augmented protein synthesis and increased degradation as revealed by the upregulation of proteins involved in ribosomes and proteasomal degradation (Figures 4A,C). On the downside of dysregulated proteins were glycolytic enzymes standing out as a major downregulated pathway (Figures 4B,C).

**Figure 5:**
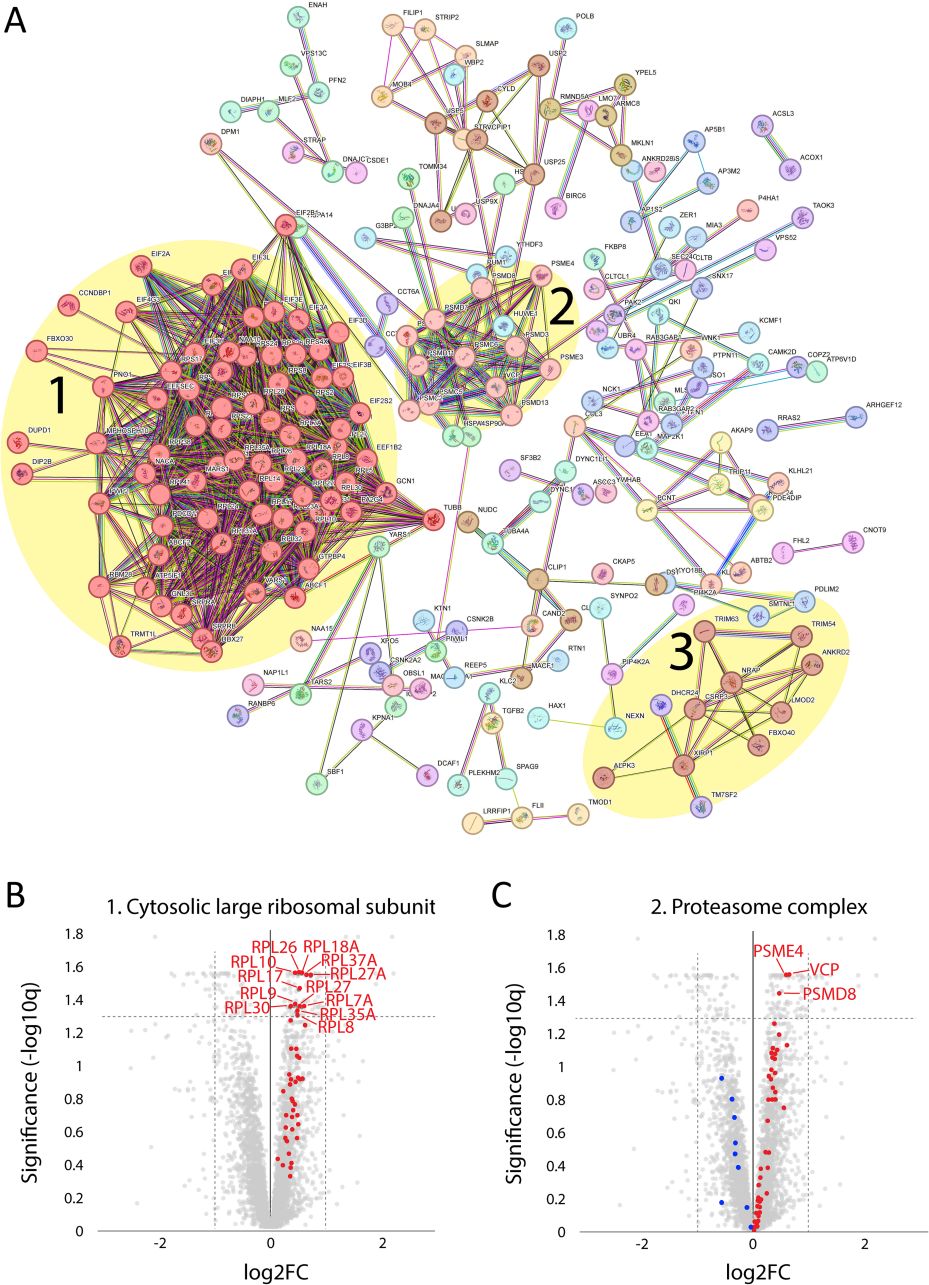
Proteomic analysis of up-regulated proteins. **(A)** From the STRING analysis, three clusters were detected and highlighted: 1, proteins involved in the cytosolic large ribosomal subunit; 2, proteins involved in the proteasome complex; 3, proteins involved in sarcomere turnover and maintenance (described in more detail in the figure 6; FDR = 0.1 Log2FC = 0.2, 256 proteins). **(B)** Volcano plot of cytosolic large ribosomal subunit proteins (GO:0022625) showing that all were up-regulated (marked in red). For those with FDR < 0.05, the gene symbols are included. **(C)** Volcano plot of proteasome complex (GO:0000502) showing most proteins are up-regulated (marked in red) and some down-regulated (marked in blue). For those with FDR < 0.05, the gene symbols are included.

**Figure 6:**
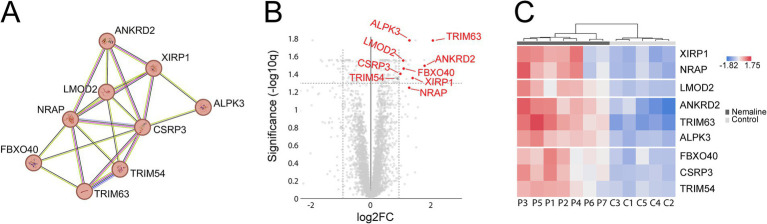
Detailed analysis of proteins included in cluster 3 from Figure 5. Proteins are given by their gene symbols. **(A)** Cluster 3 comprised of proteins involved in sarcomere turnover and maintenance. **(B)** Volcano plot showing that most of these proteins are highly and significantly upregulated. **(C)** Heat map showing the differentially expressed proteins in individual patients and controls colored according to their upregulation (red) or downregulation (blue). P, patient; C, control.

A major upregulated pathway was cytoplasmic translation with upregulated ribosomal proteins belonging to both the large and small ribosomal subunits (Figure 5A, cluster 1). The large subunit is comprised of ≈47 different proteins, most of which were identified and upregulated and 12 were significant (Figure 5B). A similar pattern was observed for the proteins comprising the cytosolic small ribosomal subunit (Supplementary Figure 1A). Another important upregulated pathway consisted of the proteasomal degradation system with several building blocks significantly increased (Figure 5A cluster 2; Figure 5C). Immunohistochemistry revealed that in a case where small fibers were generally more affected by structural alterations the proteasome 20S was accumulated in these small fibers (Supplementary Figure 2). A third cluster that was identified included the most significantly upregulated proteins (Figure 5A cluster 3; Figures 6A–C; Table 2). This cluster of nine proteins (NRAP, FBXO40, TRIM63, TRIM54, ALPK3, XIRP1, ANKRD2, LMOD2, and CSRP3) is a group of proteins that are highly expressed in muscle tissue and several of them are muscle-specific. They are important for the turnover and maintenance of sarcomere protein. The pattern was similar for both the ACTA1 and NEB patients (Figure 6C).

**Figure 7:**
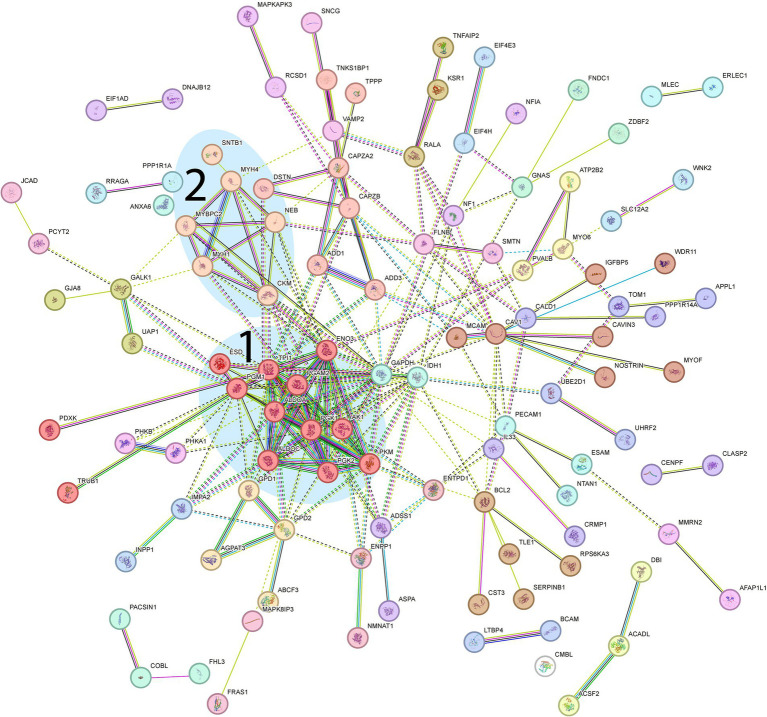
Proteomic analysis of down-regulated genes. From the STRING analysis, two clusters were detected and highlighted: 1, Proteins involved in glycolysis (further described in more detail in Figure 8); 2, Muscle-specific proteins involved in muscle contraction. (FDR = 0.1 Log2FC = 0.2, 158 proteins).

**Figure 8:**
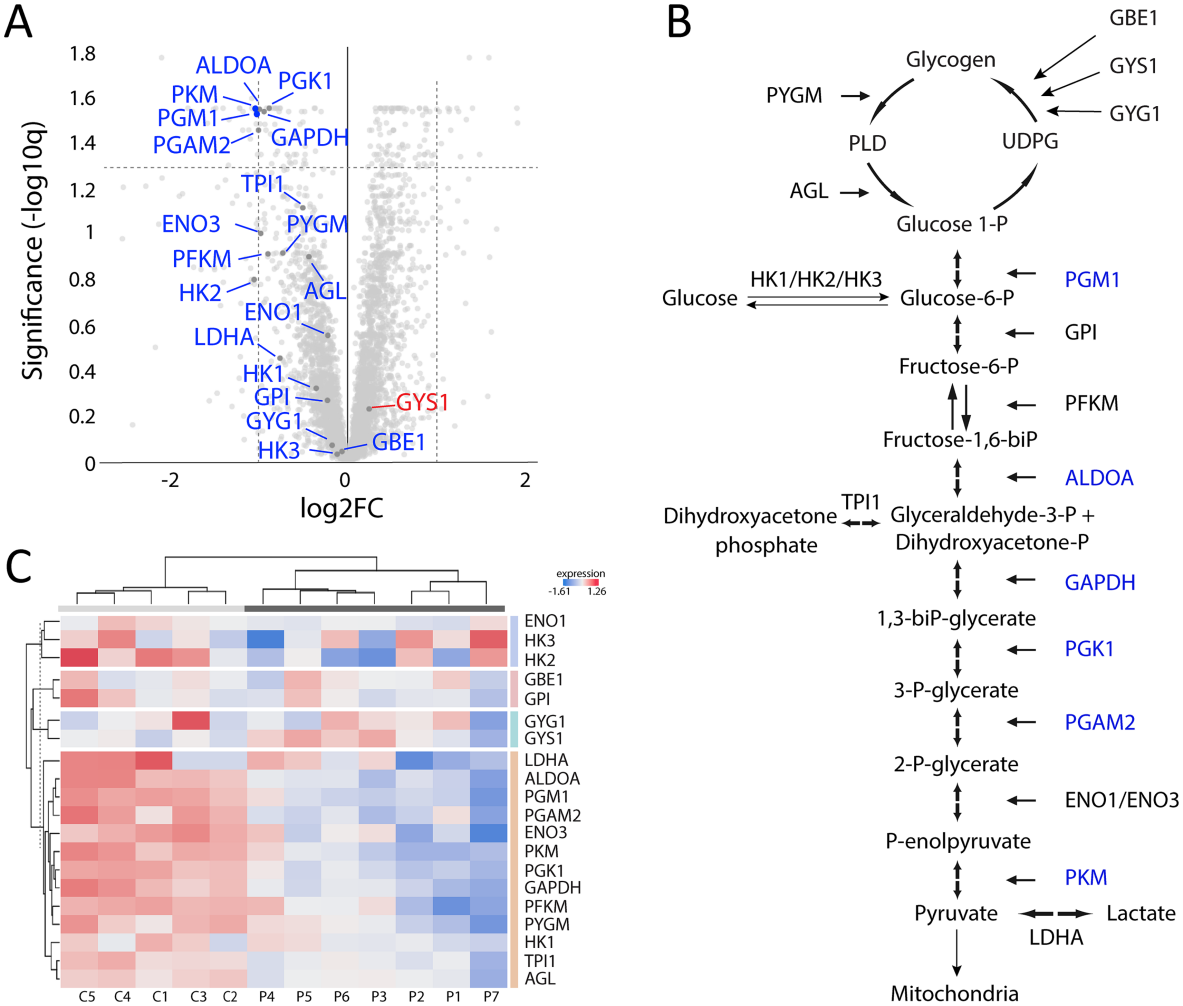
Proteomic analysis with a focus on glycogen metabolism and glycolysis. **(A)** Volcano plot of glycogen and glycolysis associated proteins showing that most of them are down-regulated (marked in blue) and that only GYS1 is up-regulated but not significantly. **(B)** Illustration of the glycogen metabolism and glycolysis with the significantly down-regulated proteins marked in blue. **(C)** Heat map showing the differentially expressed proteins in individual patients and controls, colored according to their upregulation (red) or downregulation (blue). Each column corresponds to one sample (P, patient; C, control). Dendrograms showing hierarchical clustering.

Analysis of downregulated proteins revealed the glycolysis and glycogenolysis as major dysregulated pathways where all enzymes involved were at the downside, whereas enzymes involved in the glycogen synthesis such as glycogenin-1 (GYG1), muscle glycogen synthase (GYS1) and branching enzyme (GBE1) seemed to be unaffected (Figure 7, cluster 1; Figure 8). The pattern was similar for both the ACTA1 and NEB patients (Figure 8C). Other energy metabolic pathways, especially in the mitochondria were analyzed in detail, such as the respiratory chain (Supplementary Figure 1C) but did not show any clear up- or down-regulation.

Another downregulated cluster was comprised of proteins mainly involved in muscle contraction within the sarcomeres (Figure 7, cluster 2). Among these proteins was myosin heavy chain 2X (MYH1), which is expressed in the fast type 2B (2X) fibers (14). MYH1 was the most downregulated of all proteins in the proteome.

**Figure 9:**
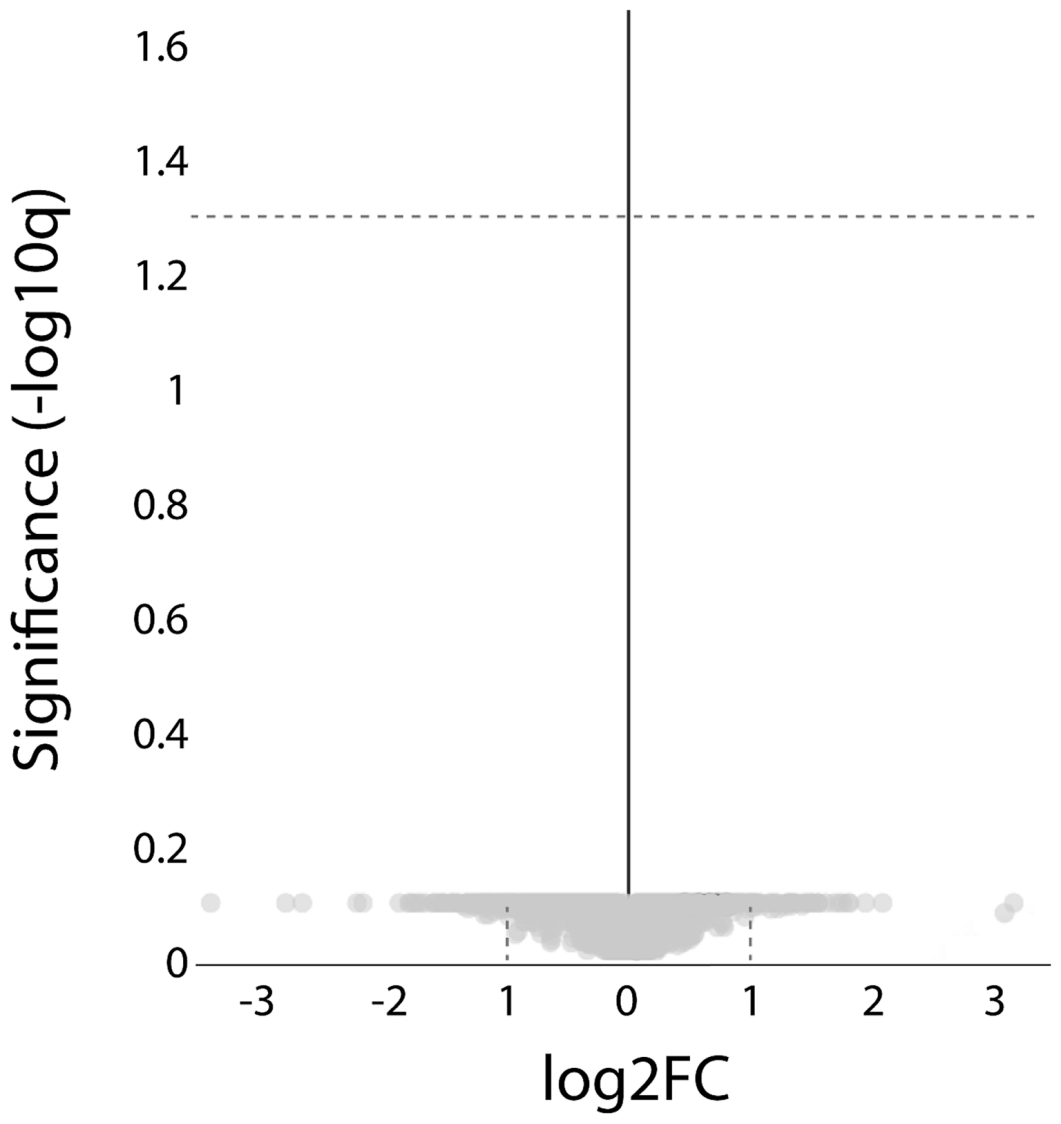
Proteomic analysis comparing the ACTA1 with the NEB cases. Volcano plot showing that no significant, differentially expressed proteins were detected (hatched line indicate FDR < 0.05).

Comparison of the results from the ACTA1 and NEB patient groups revealed no proteins with significantly different expression levels (Figure 9).

## 4 Discussion

In this study, we investigated the proteomic profile in the two most common forms of nemaline myopathy caused by either *NEB* or *ACTA1* gene variants. To reduce the influence of age on the results, only infants were included, and all analyzed muscle samples were obtained by open biopsy from living patients. We identified several dysregulated groups of proteins that may explain pathobiological events in nemaline myopathy.

Major upregulated proteins were important parts of protein synthesis and degradation, possibly indicating an increased turnover of proteins in nemaline myopathy. Ribosomal proteins were upregulated, including the building blocks of the large and small subunits of the ribosomes, indicating an increased number of ribosomes as a sign of increased protein synthesis. At the same time, the upregulated proteins, which are related to proteasomal degradation, indicate increased protein degradation, which may be related to the increased degradation or deficiency of mutated proteins in the thin filament and secondary degradation of other associated proteins in the sarcomeres. This hypothesis is supported by the loss of sarcomeres seen in large regions of affected muscle fibers, where the sarcomeres are replaced by rods (Figures 1, 2).

STRING analysis revealed that nine of the most upregulated proteins form a cluster (Figures 5A, 6). These proteins are highly expressed in muscle, and some of them are muscle-specific and involved in the structure and function of the sarcomeres.

FBXO40, TRIM54 and TRIM63 are muscle-specific E3 ubiquitin ligases. FBXO40 is a SCF E3 ubiquitin ligase involved in the regulation of the anabolic growth factor insulin like growth factor 1 (IGF1). This regulation occurs via ubiquitination and subsequent proteasomal degradation of insulin receptor substrate 1 (IRS1) in the IGF1/IRS1/PI3K/Akt pathway (15). Downregulation of Fbxo40 in mouse and pig results in muscle hypertrophy without any apparent pathology, and FBXO40 has been suggested as a target to treat human muscle disorders (16). It is therefore interesting that FBXO40 is highly upregulated both in NEB and ACTA1 associated nemaline myopathy as shown in the present study. Downregulating FBXO40 could potentially increase muscle mass and ameliorate the clinical phenotype.

TRIM54 and TRIM63 are involved in sarcomere protein regulation (17, 18), and pathogenic variants in TRIM54 and TRIM63 are associated with a protein aggregate myopathy and cardiomyopathy in mice and humans (18, 19). Our finding of upregulated sarcomere-associated ubiquitin ligases supports the concept of an increased protein turnover in nemaline myopathy. Except for FBXO40, the significantly upregulated E3 ubiquitin ligases appeared to be restricted to those involved in sarcomeric proteins, whereas others were either up- or down-regulated but not significantly (Supplementary Figure 1B).

NRAP (nebulin-anchoring protein) is a nebulin family member and thin filament chaperone that is essential for normal muscle development. It is upregulated in *KLHL41*-associated nemaline myopathy, and it has been suggested that this upregulation was secondary to KLHL41 deficiency, since NRAP is normally controlled by KLHL41 through ubiquitination-mediated proteasomal degradation (20). In nebulin-deficient zebrafish, NRAP is also upregulated and shows aberrant localization, contributing to the pathology in that nemaline myopathy model (21). Genetic ablation of NRAP restored sarcomeric disorganization, reduced protein aggregates and improved muscle function in nebulin-deficient zebrafish (21). We show that upregulation of NRAP is observed in nebulin and alpha-actin associated nemaline myopathy in humans, indicating that it may be a universal phenomenon in nemaline myopathy, and NRAP reduction may be an effective therapeutic approach in nemaline myopathy.

Alpha-kinase 3 (ALPK3) is a phosphatase that binds to M-band proteins and interacts with sequestosome-1 (SQSTM1, p62) and MURF3 (TRIM54) (22). ALPK3 genetic variants have been associated with cardiomyopathy (23). Xin (XIRP1) is a marker of muscle degeneration and is found in protein aggregates in myofibrillar myopathies (24), and is accumulated in myofibrillar lesions in kyphoscoliosis peptidase (KY) deficiency, where nemaline rods also appear (25). Ankyrin repeat protein 2 (ANKRD2) is interacting with sarcomere proteins such as titin and telethonin and is involved in the mechanical stretch response (26). Leiomodin 2 (LMOD2) is an alpha-actin binding protein essential for the assembly and length of thin filaments (27) and genetic variants are associated with cardiomyopathy (28). Cysteine-rich protein 3 (CSRP3; MLP, muscle LIM protein) is a muscle-specific, Z-disc-associated protein that is important for maintaining muscle structure and function, and it may regulate autophagy by interaction with LC3 (29). It is essential for myofibrillar organization and pathogenic variants are associated with cardiomyopathy in mice and humans (30, 31).

These nine muscle-specific upregulated proteins described above are apparently involved in muscle fiber protein turnover but their role in the pathobiology of nemaline myopathy remains elusive. It may be speculated that one or several of these proteins can act as potential therapeutic targets, which warrants further investigations.

Downregulated proteins were mainly those involved in glycogenolysis and glycolysis. All the enzymes in these pathways were on the downside and six of them had FDR < 0.05. In contrary, the enzymes essential for glycogen synthesis, i.e., glycogenin-1 (GYG1), glycogen synthase (GYS1), and branching enzyme (GBE1) seemed not to be dysregulated. Interestingly, this finding is in accordance with a previous transcriptomic analysis on a group of nemaline myopathy patients with variable and partly unknown genetic backgrounds (32). It was speculated that the increased amount of glycogen, frequently seen in muscle fibers in nemaline myopathy, may be associated with this downregulation of glycolytic enzymes (Figure 1D) (33). We found no evidence of up or downregulation of proteins essential for mitochondrial energy metabolism, which can be seen in other forms of degenerative muscle disorders such as myopathy associated with sertraline medication (34) and in primary mitochondrial diseases (35). This result may also be compared to a study on single muscle fibers in *ACTA1* and *TNNT1* associated nemaline myopathy which indicated downregulation of energy metabolism in general (36). However, a direct comparison is not possible due to different methodological approaches. Another group of proteins that was downregulated was associated with muscle contraction in the sarcomeres. One of these, the myosin heavy chain type 2X (MYH1), which is present in the fast, glycolytic type 2B (2X) fibers, is downregulated, probably as a sign of absence or reduction of pure type 2B (2X) fibers in many congenital myopathies, including nemaline myopathy. Altered fiber type composition may sometimes explain some of the protein dysregulation identified by proteomic analyses (36). However, in our cases there was type 1 fiber predominance but also type 1 fiber hypoplasia, resulting in similar volumes of glycolytic type 2A and oxidative type 1 fibers, with some variation between individuals and different regions of the muscle specimen (see also Figures 1, 2).

The major dysregulated protein pathways identified in this study exhibited a similar pattern regardless of whether the mutated gene was *ACTA1* or *NEB*. Furthermore, bioinformatic analysis revealed no proteins with significantly different expression levels between the two patient groups. This finding supports the concept that mutations in different genes may be associated with very similar phenotypes, not only from a clinical and morphological perspective but also regarding protein dysregulation. This finding may imply that there may be therapeutic approaches that can be efficient irrespective of the underlying genetic defect in nemaline myopathy.

In conclusion, we have identified some major dysregulated proteins and cellular pathways in the muscle of infants with nemaline myopathy. Our findings highlight the importance of proteomic profiling in understanding the pathobiology of nemaline myopathy. The identified proteins warrant further investigations as potential drug targets for the treatment of nemaline myopathy.

## Data Availability

The raw data supporting the conclusions of this article will be made available by the authors, without undue reservation.

## Footnotes

^1^ https://www.proteomaps.net/

^2^ https://string-db.org/
