# Supplementary material for: Proteomic analysis of nemaline myopathy in infants reveals distinct common dysregulated proteins and cellular pathways
Source: Front Neurol. 2025 Oct 3;16:1661747. doi: 10.3389/fneur.2025.1661747 (PMC12531377; doi:10.3389/fneur.2025.1661747)
Supplement: Supplementary file 1 [file Supplementary_file_1.docx]

SUPPLEMENTARY MATERIAL

Proteomic analysis of nemaline myopathy in infants reveals distinct dysregulated proteins and cellular pathways

Material and Methods

Proteomic Liquid Chromatography-Mass Spectrometry (LC-MS³) analysis - Global relative quantification

*Proteomic sample preparation*

Relative quantification was performed to compare protein expression in 7 patients with nemaline myopathy and five controls. Proteins were extracted in lysis buffer (2% sodium dodecyl sulfate, 100 mM triethylammonium bicarbonate) using using a FastPrep instrument (Matrix D, MP Biomedicals). Protein concentrations were determined using Pierce BCA Protein Assay Kit (Thermo Scientific) on a SpectraMax iD3 microplate reader (Molecular Devices). Samples were processed with a modified SP3 method (ref: doi.org/10.15252/msb.20145625). Samples (24 µg) were reduced in 10mM dithiothreitol at 56°C for 30min and alkylated in 20mM iodoacetamide at room temperature for 30min. Washed hydrophobic and hydrophilic Sera-Mag™ SpeedBeads (Carboxylate-Modified, Cytiva) were added to the samples with a bead to protein ratio of 10:1. Proteins were precipitated on the beads by ethanol (final concentration 70%) , washed with 80% ethanol and dried at room temperature. Beads were resuspended in 50 µL 100 mM TEAB and proteins were digested with Trypsin/Lys-C mix [1:25, Promega] for two hours and trypsin [1:50, Thermo Scientific] overnight. The peptide supernatants were collected, the magnetic beads were washed with 50 µL 100 mM TEAB and combined with the supernatant. Peptide concentrations were determined using Pierce™ Quantitative Fluorometric Peptide Assay (Thermo Scientific). Peptide samples (20 µg) were labelled using TMTpro 18-plex isobaric mass tagging reagents (Thermo Fisher Scientific). The labelled samples were pooled into one TMT-set and purified using HiPPR Detergent Removal Resin and Pierce peptide desalting spin columns (both Thermo Scientific), according to the manufacturer´s instructions. The TMT-set was fractionated by basic reversed-phase chromatography on a Dionex Ultimate 3000 UPLC system (Thermo Fisher Scientific). Peptide separations were performed using a reversed-phase XBridge BEH C18 column (3.5 μm, 2.1x250 mm, Waters Corporation) and a stepped gradient from 3% to 38% solvent B over 80 min followed by an increase to 80% B for 10 min at a flow of 200 µL/min. Solvent A was 25mM ammonia and solvent B was 84% acetonitrile. 140 primary fractions were automatically combined to 25 final fractions which were evaporated and reconstituted in 3% acetonitrile, 0.1% trifluoroacetic for LC-MS3 analysis.

*LC-MS3 analysis*

The fractions were analysed on an Orbitrap Lumos Tribrid mass spectrometer equipped with a FAIMS Pro ion mobility system and interfaced with an Easy-nLC1200 liquid chromatography system (all Thermo Fisher Scientific). Peptides were trapped on an Acclaim Pepmap 100 C18 trap column (100 μm x 2 cm, particle size 5 μm, Thermo Fisher Scientific) and separated on an in-house packed analytical column (35 cm x 75 μm, particle size 3 μm, Reprosil-Pur C18, Dr. Maisch) using a stepped gradient from 5% to 35% acetonitrile in 0.2% formic acid over 77 min at a flow of 300 nL/min. FAIMS Pro was alternating between the compensation voltages (CV) of -50 and -70, and the same data-dependent settings were used at both CVs. The precursor ion mass spectra were acquired at a resolution of 120 000 and an m/z range of 375-1375. Using a cycle time of 1.5 seconds the most abundant precursors with charges 2–7 were isolated with an m/z window of 0.7 and fragmented by collision induced dissociation (CID) at 35%. Fragment spectra were recorded in the ion trap at Rapid scan rate. Dynamic exclusion was set to 60 sec. The ten most abundant MS2 fragment ions were isolated using multi-notch isolation for further MS3 fragmentation. MS3 fragmentation was performed using higher-energy collision dissociation (HCD) at 55% and the MS3 spectra were recorded in the Orbitrap at 50 000 resolution and an m/z range of 100–500.

*Proteomic data analysis*

Raw files were processed and analyzed with Proteome Discoverer (Ver 3.0, Thermo Scientific) The data was matched against *Homo Sapiens* SwissProt database (20421 entries, Jan 2025) using Sequest as a search engine with a precursor tolerance of 5 ppm and a fragment ion tolerance of 0.6 Da. Tryptic peptides were accepted with 1 missed cleavage. Methionine oxidation was set as a variable modifications and cysteine carbamidomethylation, TMTpro on lysine and peptide N-termini were set as fixed modifications. Percolator was used for PSM validation with a strict FDR threshold of 1%. For quantification TMT reporter ions were identified in the MS3 HCD spectra with 3 mmu mass tolerance and the TMT reporter intensity values for each sample were normalized on the total peptide amount. The SPS threshold was set to 65%, a Sequest HT threshold score of 2 was chosen. Only unique peptides were used for relative quantification and proteins were required to pass a protein FDR of 5%.

Results

**Supplementary Table 1.** 183 significant down- and up-regulated proteins were identified (FDR < 0.05)

| **Gene symbol** | **Description** | **log2FC** | **FDR** |
| --- | --- | --- | --- |
| MYH1 | myosin heavy chain 1 | -3.98 | 0.03 |
| GJA8 | gap junction protein alpha 8 | -3.81 | 0.03 |
| RBM20 | RNA binding motif protein 20 | -2.09 | 0.02 |
| PTER | phosphotriesterase related | -1.79 | 0.03 |
| MYH4 | myosin heavy chain 4 | -1.74 | 0.03 |
| ACADL | acyl-CoA dehydrogenase long chain | -1.57 | 0.03 |
| ART3 | ADP-ribosyltransferase 3 (inactive) | -1.50 | 0.03 |
| BCL2 | BCL2 apoptosis regulator | -1.48 | 0.03 |
| DBI | diazepam binding inhibitor, acyl-CoA binding protein | -1.40 | 0.03 |
| IGFBP5 | insulin like growth factor binding protein 5 | -1.34 | 0.05 |
| TPPP | tubulin polymerization promoting protein | -1.33 | 0.03 |
| CMBL | carboxymethylenebutenolidase homolog | -1.21 | 0.03 |
| EGFLAM | EGF like, fibronectin type III and laminin G domain | -1.21 | 0.03 |
| CA14 | carbonic anhydrase 14 | -1.18 | 0.03 |
| TLE1 | TLE family member 1, transcriptional corepressor | -1.17 | 0.03 |
| CRADD | CASP2 and RIPK1 domain containing adaptor with death domain | -1.11 | 0.03 |
| AK1 | adenylate kinase 1 | -1.09 | 0.04 |
| GPD2 | glycerol-3-phosphate dehydrogenase 2 | -1.07 | 0.02 |
| WDR11 | WD repeat domain 11 | -1.07 | 0.03 |
| PLCL1 | phospholipase C like 1 (inactive) | -1,03 | 0.03 |
| ADSS1 | adenylosuccinate synthase 1 | -1.02 | 0.03 |
| PGM1 | phosphoglucomutase 1 | -1.02 | 0.03 |
| PKM | pyruvate kinase M1/2 | -1.01 | 0.03 |
| GPD1 | glycerol-3-phosphate dehydrogenase 1 | -1.01 | 0.04 |
| PGAM2 | phosphoglycerate mutase 2 | -1.00 | 0.03 |
| ALDOA | aldolase, fructose-bisphosphate A | -0.99 | 0.03 |
| AGPAT3 | 1-acylglycerol-3-phosphate O-acyltransferase 3 | -0.98 | 0.03 |
| CKM | creatine kinase, M-type | -0.97 | 0.04 |
| GAPDH | glyceraldehyde-3-phosphate dehydrogenase | -0.94 | 0.03 |
| ALDOC | aldolase, fructose-bisphosphate C | -0.93 | 0.03 |
| CRIP2 | cysteine rich protein 2 | -0.92 | 0.03 |
| INPP1 | inositol polyphosphate-1-phosphatase | -0.91 | 0,03 |
| PGK1 | phosphoglycerate kinase 1 | -0.88 | 0.03 |
| NUDT15 | nudix hydrolase 15 | -0.86 | 0.04 |
| PHKA1 | phosphorylase kinase regulatory subunit alpha 1 | -0.85 | 0.05 |
| ESAM | endothelial cell adhesion molecule | -0.85 | 0.03 |
| PGK2 | phosphoglycerate kinase 2 | -0.82 | 0.03 |
| EIF4H | eukaryotic translation initiation factor 4H | -0.82 | 0.03 |
| TRUB1 | TruB pseudouridine synthase family member 1 | -0.81 | 0.03 |
| PHKB | phosphorylase kinase regulatory subunit beta | -0.81 | 0.04 |
| DIP2C | disco interacting protein 2 homolog C | -0.79 | 0.04 |
| SLC12A2 | solute carrier family 12 member 2 | -0.79 | 0.03 |
| NFIA | nuclear factor I A | -0.78 | 0.04 |
| PLXDC2 | plexin domain containing 2 | -0.77 | 0.03 |
| JCAD | junctional cadherin 5 associated | -0.76 | 0.03 |
| PALM2AKAP2 | PALM2 and AKAP2 fusion | -0.75 | 0.03 |
| ATP2B2 | ATPase plasma membrane Ca2+ transporting 2 | -0,72 | 0.05 |
| MAPKAPK3 | MAPK activated protein kinase 3 | -0.70 | 0.04 |
| MYOF | myoferlin | -0.68 | 0.04 |
| NTAN1 | N-terminal asparagine amidase | -0.66 | 0.03 |
| CAVIN3 | caveolae associated protein 3 | -0.65 | 0.04 |
| IDH1 | isocitrate dehydrogenase (NADP(+)) 1 | -0.65 | 0.05 |
| DSTN | destrin, actin depolymerizing factor | -0.63 | 0.05 |
| MLEC | malectin | -0.60 | 0.04 |
| IL33 | interleukin 33 | -0.59 | 0.05 |
| CDV3 | CDV3 homolog | -0.59 | 0.04 |
| TIMM10 | translocase of inner mitochondrial membrane 10 | -0,58 | 0.04 |
| EIF4E3 | eukaryotic translation initiation factor 4E family, member 3 | -0,57 | 0.04 |
| RGMA | repulsive guidance molecule BMP co-receptor a | -0.53 | 0.03 |
| VAMP2 | vesicle associated membrane protein 2 | -0.52 | 0.03 |
| NOVA1 | NOVA alternative splicing regulator 1 | -0.50 | 0.03 |
| RPS6KA3 | ribosomal protein S6 kinase A3 | -0.49 | 0.05 |
| CHRAC1 | chromatin accessibility complex subunit 1 | -0.48 | 0.05 |
| FLNB | filamin B | -0.48 | 0.03 |
| RCSD1 | RCSD domain containing 1 | -0.48 | 0.04 |
| CGGBP1 | CGG triplet repeat binding protein 1 | -0.48 | 0.04 |
| GALK1 | galactokinase 1 | -0.47 | 0.05 |
| PECAM1 | platelet and endothelial cell adhesion molecule 1 | -0.37 | 0.03 |
| RRAGA | Ras related GTP binding A | -0.36 | 0.03 |
| ABCF3 | ATP binding cassette subfamily F member 3 | -0.36 | 0.04 |
| SRRM2 | serine/arginine repetitive matrix 2 | -0.30 | 0.03 |
| YWHAB | tyrosine 3-monooxygenase/tryptophan 5-monooxygenas activation protein, beta | 0.23 | 0.05 |
| EIF3A | eukaryotic translation initiation factor 3 subunit A | 0.25 | 0.03 |
| HSPA14 | heat shock protein family A (Hsp70) member 14 | 0.25 | 0.04 |
| VPS52 | VPS52 subunit of GARP complex | 0.27 | 0.03 |
| RPS28 | ribosomal protein S28 | 0,27 | 0.05 |
| DIP2B | disco interacting protein 2 homolog B | 0.30 | 0.04 |
| YPEL5 | yippee like 5 | 0.31 | 0.03 |
| EIF3CL | eukaryotic translation initiation factor 3 subunit C like | 0.32 | 0.03 |
| PAK2 | p21 (RAC1) activated kinase 2 | 0.32 | 0.03 |
| SPAG9 | sperm associated antigen 9 | 0.33 | 0.04 |
| RPS11 | ribosomal protein S11 | 0.33 | 0.05 |
| SEC24C | SEC24 homolog C, COPII coat complex component | 0.33 | 0.03 |
| RAB3GAP2 | RAB3 GTPase activating non-catalytic protein subunit 2 | 0.35 | 0.04 |
| EIF3L | eukaryotic translation initiation factor 3 subunit L | 0.35 | 0.03 |
| RPL30 | ribosomal protein L30 | 0.36 | 0.04 |
| DYNC1LI1 | dynein cytoplasmic 1 light intermediate chain 1 | 0.36 | 0.03 |
| YTHDF3 | YTH N6-methyladenosine RNA binding protein F3 | 0.36 | 0.03 |
| EIF3D | eukaryotic translation initiation factor 3 subunit D | 0.37 | 0.04 |
| TRMT1L | tRNA methyltransferase 1 like | 0,37 | 0.03 |
| VARS1 | valyl-tRNA synthetase 1 | 0.37 | 0.03 |
| RPS26 | ribosomal protein S26 | 0.38 | 0.05 |
| CSNK2B | casein kinase 2 beta | 0.38 | 0.05 |
| EEFSEC | eukaryotic elongation factor, selenocysteine-tRNA specific | 0.38 | 0.04 |
| TRIP11 | thyroid hormone receptor interactor 11 | 0.41 | 0.03 |
| ABCF2 | ATP binding cassette subfamily F member 2 | 0.41 | 0.03 |
| RPL9 | ribosomal protein L9 | 0.43 | 0.04 |
| RPS23 | ribosomal protein S23 | 0.43 | 0.03 |
| PLEKHM2 | pleckstrin homology and RUN domain containing M2 | 0.44 | 0.03 |
| MARS1 | methionyl-tRNA synthetase 1 | 0.44 | 0.05 |
| CLTB | clathrin light chain B | 0.44 | 0.04 |
| FKBP8 | FKBP prolyl isomerase 8 | 0.44 | 0.04 |
| ARMC8 | armadillo repeat containing 8 | 0.45 | 0.03 |
| GCN1 | GCN1 activator of EIF2AK4 | 0.45 | 0.04 |
| PSMD8 | proteasome 26S subunit, non-ATPase 8 | 0.47 | 0.04 |
| DCAF1 | DDB1 and CUL4 associated factor 1 | 0.47 | 0.04 |
| RPL35A | ribosomal protein L35a | 0.47 | 0.05 |
| MKLN1 | muskelin 1 | 0.49 | 0.03 |
| RPL8 | ribosomal protein L8 | 0.49 | 0.05 |
| CSDE1 | cold shock domain containing E1 | 0.50 | 0.03 |
| RPL10 | ribosomal protein L10 | 0,50 | 0.03 |
| SBF1 | SET binding factor 1 | 0.50 | 0.05 |
| AKAP9 | A-kinase anchoring protein 9 | 0.50 | 0.03 |
| RPL27 | ribosomal protein L27 | 0.50 | 0.04 |
| RPL17 | ribosomal protein L17 | 0.51 | 0.03 |
| CSNK2A2 | casein kinase 2 alpha 2 | 0.51 | 0.03 |
| P4HA1 | prolyl 4-hydroxylase subunit alpha 1 | 0.51 | 0,05 |
| RPL31 | ribosomal protein L31 | 0.52 | 0.03 |
| SF3B2 | splicing factor 3b subunit 2 | 0.52 | 0.03 |
| MPHOSPH10 | M-phase phosphoprotein 10 | 0.54 | 0.03 |
| PTPN11 | protein tyrosine phosphatase non-receptor type 11 | 0.55 | 0.05 |
| CLASP1 | cytoplasmic linker associated protein 1 | 0.56 | 0.04 |
| RPL26 | ribosomal protein L26 | 0.56 | 0.03 |
| RPL7A | ribosomal protein L7a | 0.57 | 0.04 |
| RPL18A | ribosomal protein L18a | 0.57 | 0.03 |
| SRPRA | SRP receptor subunit alpha | 0.57 | 0.03 |
| LMO7 | LIM domain 7 | 0.58 | 0.04 |
| RHOT2 | ras homolog family member T2 | 0.58 | 0.03 |
| RPS24 | ribosomal protein S24 | 0.60 | 0.05 |
| PSME4 | proteasome activator subunit 4 | 0.60 | 0.03 |
| PCNT | pericentrin | 0.61 | 0.04 |
| HSP90AB1 | heat shock protein 90 alpha family class B member 1 | 0.62 | 0.04 |
| HSPA4 | heat shock protein family A (Hsp70) member 4 | 0.62 | 0.05 |
| KPNA1 | karyopherin subunit alpha 1 | 0.63 | 0.04 |
| VCP | valosin containing protein | 0.63 | 0.03 |
| PDCD11 | programmed cell death 11 | 0.64 | 0.03 |
| FLII | FLII actin remodeling protein | 0.65 | 0.03 |
| RPL37A | ribosomal protein L37a | 0.66 | 0.03 |
| LRRFIP1 | LRR binding FLII interacting protein 1 | 0.68 | 0.04 |
| RPL27A | ribosomal protein L27a | 0.73 | 0.03 |
| HSPB6 | heat shock protein family B (small) member 6 | 0.76 | 0.05 |
| TWF2 | twinfilin actin binding protein 2 | 0.79 | 0.05 |
| PIWIL1 | piwi like RNA-mediated gene silencing 1 | 0.80 | 0.05 |
| FDXR | ferredoxin reductase | 0.80 | 0.04 |
| CCNDBP1 | cyclin D1 binding protein 1 | 0.81 | 0.03 |
| KLC2 | kinesin light chain 2 | 0.81 | 0.03 |
| RRAS2 | RAS related 2 | 0.81 | 0.04 |
| PTPN1 | protein tyrosine phosphatase non-receptor type 1 | 0.84 | 0.03 |
| PFN2 | profilin 2 | 0.86 | 0.05 |
| FILIP1 | filamin A interacting protein 1 | 0.86 | 0.04 |
| PRDM2 | PR/SET domain 2 | 0.86 | 0.04 |
| TMOD1 | tropomodulin 1 | 0.87 | 0.04 |
| CLTCL1 | clathrin heavy chain like 1 | 0.87 | 0.03 |
| AP5B1 | adaptor related protein complex 5 subunit beta 1 | 0.88 | 0.03 |
| CLIP1 | CAP-Gly domain containing linker protein 1 | 0.88 | 0.03 |
| CYLD | CYLD lysine 63 deubiquitinase | 0.88 | 0.03 |
| MAP3K20 | mitogen-activated protein kinase kinase kinase 20 | 0.88 | 0.03 |
| SYNPO2 | synaptopodin 2 | 0.88 | 0.05 |
| ARMCX4 | armadillo repeat containing X-linked 4 | 0.90 | 0.03 |
| DST | dystonin | 0.90 | 0.04 |
| SMTNL1 | smoothelin like 1 | 0.92 | 0.03 |
| TRIM54 | tripartite motif containing 54 | 0.94 | 0.04 |
| ZER1 | zyg-11 related cell cycle regulator | 0.95 | 0.05 |
| DNAJA4 | DnaJ heat shock protein family (Hsp40) member A4 | 0.97 | 0.04 |
| DIAPH1 | diaphanous related formin 1 | 1.00 | 0.03 |
| CAMK2D | calcium/calmodulin dependent protein kinase II delta | 1.03 | 0.03 |
| FBXO40 | F-box protein 40 | 1.05 | 0.04 |
| TM7SF2 | transmembrane 7 superfamily member 2 | 1.13 | 0.03 |
| SLMAP | sarcolemma associated protein | 1.14 | 0.03 |
| LMOD2 | leiomodin 2 | 1.14 | 0.03 |
| CSRP3 | cysteine and glycine rich protein 3 | 1.16 | 0.03 |
| OBSL1 | obscurin like cytoskeletal adaptor 1 | 1.23 | 0.03 |
| KLHL21 | kelch like family member 21 | 1.25 | 0.03 |
| ICMT | isoprenylcysteine carboxyl methyltransferase | 1.27 | 0.04 |
| MAP3K7CL | MAP3K7 C-terminal like | 1.33 | 0.03 |
| ALPK3 | alpha kinase 3 | 1.37 | 0.02 |
| HOMER2 | homer scaffold protein 2 | 1.40 | 0.03 |
| XIRP1 | xin actin binding repeat containing 1 | 1.48 | 0.04 |
| CYP2J2 | cytochrome P450 family 2 subfamily J member 2 | 1.59 | 0.02 |
| INPP4B | inositol polyphosphate-4-phosphatase type II B | 1.90 | 0.03 |
| ANKRD2 | ankyrin repeat domain 2 | 1.91 | 0.03 |
| TRIM63 | tripartite motif containing 63 | 2.20 | 0.02 |
| ASXL2 | ASXL transcriptional regulator 2 | 2.23 | 0.03 |

Gene symbols are included for the encoded protein


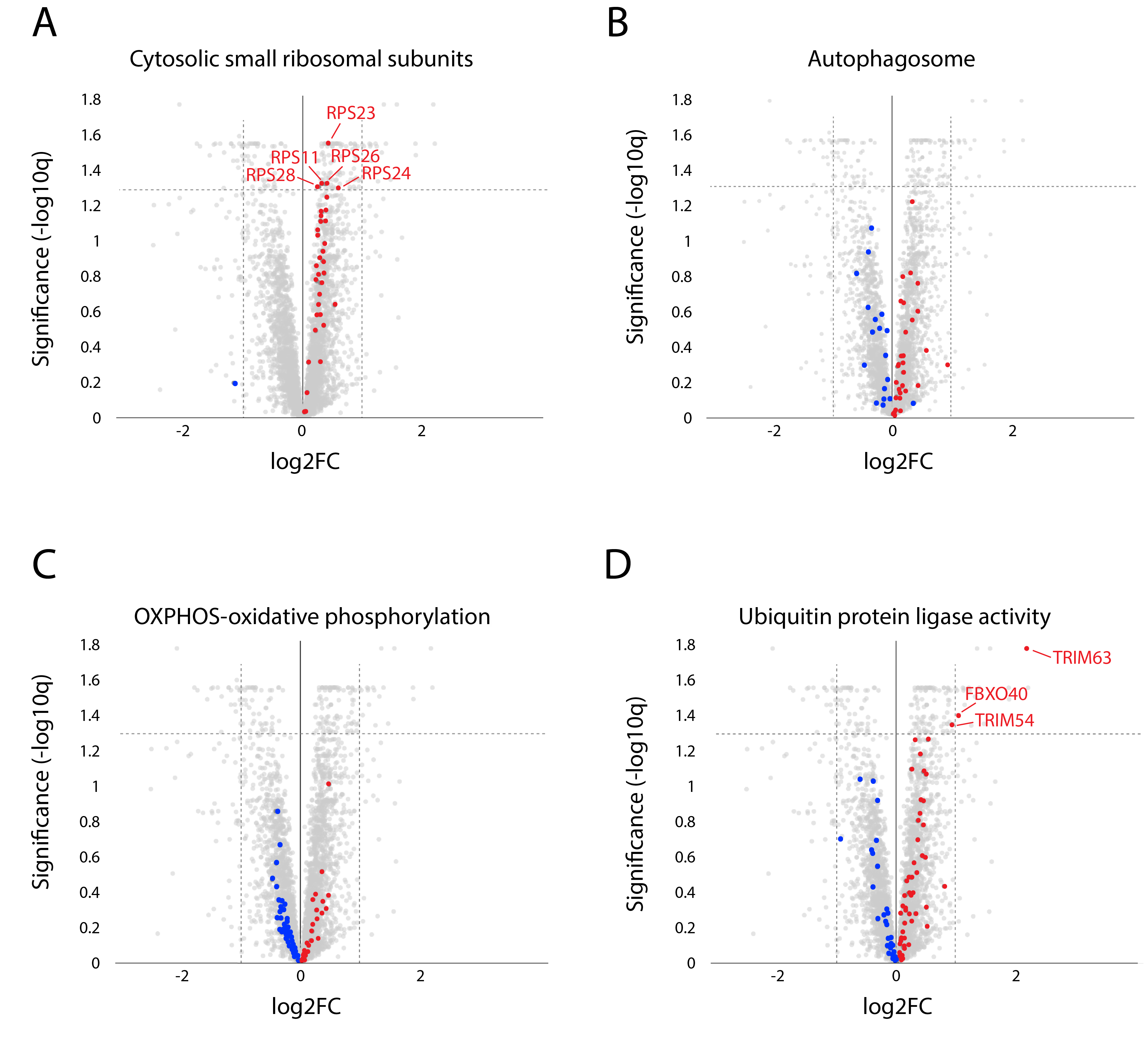


Supplementary Fig. 1. Volcano plots of different pathways with down-regulated proteins marked in blue and up-regulated marked in red and for those with FDR < 0.05 the gene symbols are included. A) Cytosolic large ribosomal subunit proteins (GO_0022627) shows that almost all proteins were up-regulated. B) Autophagosome (GO:0005776) showing non-significantly down- and up-regulated proteins, C) Oxidative phosphorylation (subunits of complex I-V of the respiratory chain) showing non-significantly down- and up-regulated proteins. D) Ubiquitin protein ligase activity (GO:0061630) shows that it was both down- and up-regulated proteins. The significantly upregulated proteins are associated with sarcomere turnover.
